# Relationship between p16/ki67 immunoscores and *PAX1*/*ZNF582* methylation status in precancerous and cancerous cervical lesions in high-risk HPV-positive women

**DOI:** 10.1186/s12885-024-12920-4

**Published:** 2024-09-20

**Authors:** Haijun Luo, Yixiang Lian, Hong Tao, Yan Zhao, Zhigan Wang, Jing Zhou, Zirong Zhang, Shali Jiang

**Affiliations:** 1 Department of Pathology, The Affiliated Changsha Central Hospital, Hengyang Medical School, University of South China, Changsha, 410004 China; 2 Department of Medical Statistics, Hunan Hoomya Gene Technology Co., Ltd., Changsha, 410205 China

**Keywords:** *PAX1* methylation, *ZNF582* methylation, p16, Ki67, Immunoscore, CIN

## Abstract

**Background:**

The risk of cervical cancer progression in high-risk human papillomavirus (HR-HPV)-positive women is associated with cervical lesion severity and molecular heterogeneity. Classification systems based on p16 and Ki67 expression cumulative scores (0–3 each)—p16/Ki67 collectively known as an immunoscore [IS]—are an accurate and reproducible method for grading cervical intraepithelial neoplasia (CIN) lesions. Meanwhile, DNA methylation is an early event in the development of cervical cancer. Hence, this study evaluated the relationship among CIN, p16/Ki-67 IS, and *PAX1*/*ZNF582* methylation.

**Methods:**

In this study, 414 HPV-positive paraffin-embedded specimens were collected, and *PAX1*/*ZNF582* methylation and the p16/ki67 IS were determined. A total of 43 invalid samples were excluded and 371 were included in the statistical analyses. There were 103 cervicitis, 95 CIN1, 71 CIN2, 89 CIN3, and 13 squamous cell carcinoma (SCC) cases. The association between *PAX1*/*ZNF582* methylation and p16/Ki6 immunohistochemical staining scores was analyzed.

**Results:**

The ΔCp of *PAX1*^*m*^ (*PAX1* methylation) and *ZNF582*^*m*^ (*ZNF582* methylation) decreased with cervical lesion severity (Cuzick trend test, all *P* < 0.001). The severity of the cervical lesions and p16, Ki67, and p16/Ki67 IS showed an increasing trend (Multinomial Cochran-Armitage trend test, all *P* < 0.001). The prevalence of *PAX1*^*m*^/*ZNF582*^*m*^ increased with an increase in the IS of p16, Ki67, and p16/Ki67 (Cochran-Armitage trend test, all *P* < 0.001). In cervical SCC, the IS was 5–6, and the *PAX1*^*m*^/*ZNF582*^*m*^ was positive. Meanwhile, heterogeneity was observed in CIN lesions: 10 cases had an IS of 3–4 and were *PAX1*^*m*^/*ZNF582*^*m*^-positive in ≤ CIN1; 1 case had an IS of 0–2 and was *PAX1*^*m*^/*ZNF582*^*m*^-positive in CIN2/3.

**Conclusions:**

Significant heterogeneity was observed in CIN lesions for p16 and Ki67 immunohistochemical staining scores and *PAX1*/*ZNF582* methylation. This may help clinicians personalize the management of CIN based on the predicted short-term risk of cancer progression, minimizing the rate of missed CIN1 diagnoses and incorrect treatment of CIN2/3.

**Supplementary Information:**

The online version contains supplementary material available at 10.1186/s12885-024-12920-4.

## Background

Persistent infection with high-risk human papillomavirus (HR-HPV) is the main cause of cervical cancer. Most patients with grade 3 cervical intraepithelial neoplasia (CIN) develop cervical cancer [1]. CIN lesions can be classified into grades 1, 2, and 3 based on the area in which the normal epithelium has been replaced by atypical epithelium, determined by histological analysis, and the area in which abnormal cells are atypical. However, classical histological grading of CIN remains subjective, with considerable inter- and intra-observer variation and moderate reproducibility [2]. The pathological results of colposcopy biopsies are also inconsistent with those after coning, with ∼ 10% of CIN3 coning pathological results diagnosed as cervical cancer [3].

The accurate evaluation of cervical lesions is critical to selecting clinical treatments. The current standard practice is to treat CIN3 and most CIN2 lesions with macrocyclic or conical resection of the cervical transformation zone to prevent cervical cancer development. Spontaneous regression of CIN2 (40–50%) and CIN3 (∼ 30%) lesions frequently occurs [4, 5]. Therefore, excision of all high-grade cervical lesions (CIN2/3) could lead to overtreatment. Clinically, cervicitis or CIN1, as determined through colposcopy biopsy, can progress to cervical cancer within a short period without developing CIN2 and CIN3 [6, 7]. In these patients, cervical cancer may develop during normal follow-up.

CIN is a heterogeneous lesion categorized by the production of new viral particles (pCIN) or transforming CIN lesions (tCIN) that integrate HPV DNA into host cells. Biomarkers can distinguish between pCIN and tCIN, with the latter considered to have a higher risk of rapid progression to cervical cancer [8]. p16 is an important biomarker for detecting transforming HPV and is used as a surrogate indicator of HPV E7 transforming activity [8]. Currently, p16 immunohistochemical staining is used to morphologically identify suspected cases of low- and high-grade CIN [2]. Additionally, Ki67 is a marker of cell cycle activity and CIN classification [9, 10]. The co-expression of p16 and Ki67 is associated with cellular dysfunction and occurs in dysplastic cervical cells. Hence, their expression is considered a useful tool for detecting progressing cervical disease [11, 12]. In fact, classification systems based on p16 (0–3) and Ki67 (0–3) expression cumulative scores (i.e., the immunoscore) have proven more accurate and reproducible for grading CIN than hematoxylin–eosin (H&E) staining alone. The cumulative score also reduces the diagnosis rate of grade 2 CIN with inconsistent clinical management [13].

DNA methylation in the promoter regions of tumor suppressor genes precedes cervical cancer development [8, 14, 15]. Several studies have shown that methylation markers can be used to identify transforming CIN lesions and predict CIN lesion degeneration or progression [16–23]. Additionally, HR-HPV infections that persist for at least 5 years are associated with CIN2/3 disease with cancer-like methylation patterns, suggesting a higher risk of short-term cervical cancer progression [8, 24]. Thus, combining DNA methylation analysis with p16 and Ki67 immunohistochemical staining may provide a biomarker phenotype to improve clinical guidance for women with CIN and prevent overtreatment of reversible lesions and potential obstetric complications.

This study evaluates the relationship among CIN, p16/Ki67 immunoscores, and *PAX1*/*ZNF582* methylation. To assess CIN heterogeneity, all cases were stratified according to the p16/Ki-67 immunoscores and PAX1/ZNF582 methylation status of patients. The primary objective of this study was to provide insights to further the development of individual treatment regimens for CIN based on the predicted cancer progression risk.

## Methods

### Study population and sample collection

In this retrospective study, HR-HPV-positive patients with cervicitis, CIN1, CIN2, CIN3, or squamous cell carcinoma (SCC) who were diagnosed via histopathological analysis (colposcopy biopsy) at Changsha Central Hospital from October 2022 to July 2023 were included in the study population. The inclusion criteria included (1) HR-HPV-positive and (2) agreeing to participate and signing informed consent. The exclusion criteria included (1) women with a previous cancer history, (2) previous treatment of CIN or cancer, (3) immunodeficiency, and (4) HPV vaccination history.

Paraffin-embedded biopsies were cut into five slices, two of which were used to evaluate p16 and Ki67 expression. The remaining three sections were tested for *PAX1* and *ZNF582* methylation. This study was approved by the Medical Ethics Committee of the Affiliated Changsha Central Hospital, Hengyang Medical School, University of South China (approval number: 2022-S0165), and informed consent was obtained from all patients.

### HR-HPV testing

HR-HPV was detected using the HPV gene array test kit (HybriBio Ltd., Guangzhou, China), including 14 h-HPV genotypes (HPV16, 18, 31, 33, 35, 39, 45, 51, 52, 56, 58, 59, 66, and 68) and genotyped according to the manufacturer’s instructions.

### Scoring of p16/Ki67

Two pathologists, blinded to patient clinical information and DNA methylation results, performed p16 and Ki67 immunohistochemical staining of the paraffin sections. If the scores of the two pathologists differed, a third expert was asked to score the results, and the preference of the final third expert prevailed.

For p16, a score of 0 was defined as positive cells or small cell clusters without p16 positive reaction or local dispersion; 1 represented a diffusively positive response limited to the lower third of the epithelium, 2 represented positive responses only in the lower half of the epithelium, and 3 represented responses throughout the epithelial thickness. Immunohistochemical staining for nuclear Ki67 was performed in keratinocytes and as scored as follows: a score of 0 indicated a normal expression pattern and scattered staining of nuclei in the basal layers; scores of 1, 2, and 3 were defined as having expression distributed in the lower third, lower half, and more than two-thirds of the epithelium, respectively. The cumulative immunohistochemical staining scores for p16/Ki67 (ranging from 0 to 6) were designated the “immune score” (IS), which could be categorized into three groups: an IS of 5–6 was associated with CIN3 + lesions, 3–4 was associated with CIN2 lesions, and 0–2 was associated with CIN1 lesions.

### Measurement of PAX1/ZNF582 methylation

The methylation status of *PAX1* and *ZNF582* was evaluated using a cervical cancer gene methylation test kit (Hoomya, Changsha, China) on the Cobas 480 II PCR platform. The *COL2A1* gene was used as an internal reference gene; a Cp value > 35 indicated an insufficient sample size and an invalid methylation test. The methylation value was obtained by subtracting the Cp value of *COL2A1* from that of the target gene to calculate the difference (ΔCp). The lower the ΔCp value of *PAX1*^*m*^ and *ZNF582*^*m*^, the higher the methylation level.

### Statistical analysis

Data were analyzed using R V 4.3.2. The CIN grades were original local diagnoses used in the hospital. Continuous variables were recorded as medians [IQR], and categorical variables were recorded as counts (%). The distribution of methylation (ΔCp), determined by pathology and IS, was mapped using the ggpubr package. The ggplot2 package was used to create the stacking plot of the IS and pathology results. The pROC package was used to map the receiver operating characteristics (ROC) curve for the diagnostic performance of DNA methylation for CIN3+ (Supplementary Fig. 1). Following the Youden index of the ROC maximization principle, the cut-off value for *PAX1*^*m*^ was 11.9; hence, the text was deemed positive when ΔCp ≤ 11.9 and negative when ΔCp > 11.9. The cut-off value for *ZNF582*^*m*^ was 10.8. Cuzick’s test was performed to identify trends between continuous and ordered categorical variables using the PMCMRplus package. Trends between two ordered categories were analyzed using the DescTools and multiCA packages. A mosaic plot was constructed using the VCD package. Two-sided *P* values < 0.05 were considered statistically significant.

## Results

### Case series

In this study, 414 paraffin specimens from HR-HPV-positive patients were collected from the pathology department, of which 33 were invalid for the PAX1m assessment, and 43 were invalid for the ZNF582m assessment. Thus, 43 cases were excluded, and 371 patients were included in the statistical analysis, with a median age of 42 years (IQR: 32–54). Supplementary Table 1 shows the cytology results of p16, Ki67, PAX1m, and ZNF582m. There were 103 cases of cervicitis (27.8%), 95 cases of CIN1 (25.6%), 71 cases of CIN2 (19.1%), 89 cases of CIN3 (24.0%), and 13 of cervical SCC (3.5%).

### DNA methylation increased with increased cervical lesion severity

**Fig. 1:**
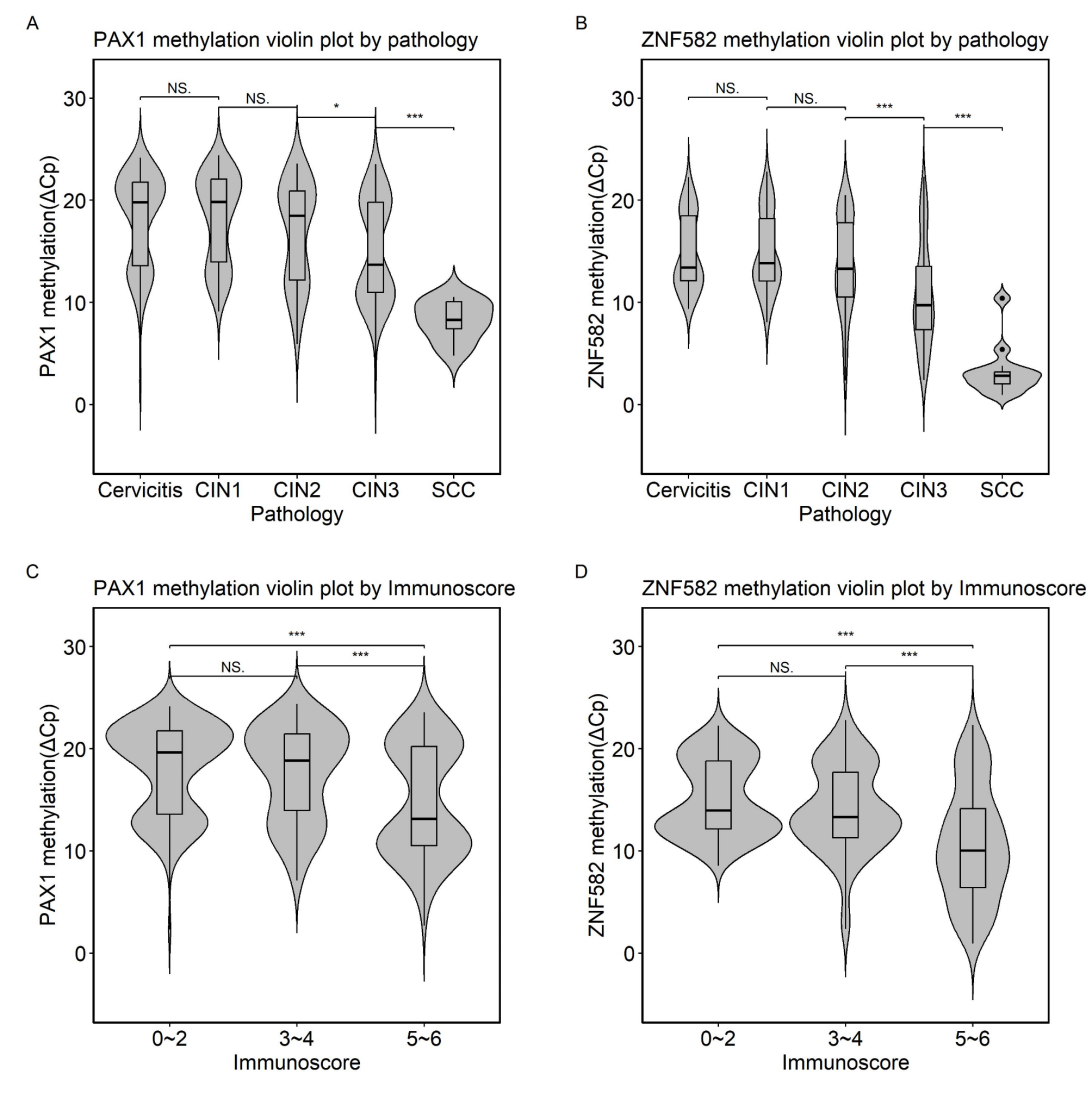
Violin plot of *PAX1/ZNF582* methylation distribution in cervical lesions and p16/Ki67 immune scores. A, Distribution of PAX1^m^ (ΔCp)  grouped according to pathology results. B,  Distribution of ZNF582^m^ (ΔCp)  grouped according to pathology results.C, Distribution of PAX1^m^ (ΔCp)  grouped according to IS results. D, Distribution of ZNF582^m^ (ΔCp)  grouped according to IS results. NS., no significance; * *P* < 0.05; ** *P* < 0.01; *** *P* < 0.001

The ΔCp values for *PAX1*^*m*^ and *ZNF582*^*m*^ decreased with increasing lesion degree (Cuzick trend test, all *P* < 0.001, Table 1). The distribution of *PAX1*^*m*^ and *ZNF582*^*m*^ in cervical lesions significantly differed between CIN2 and CIN3 (all *P* < 0.05; Fig. 1A and B). The severity of cervical lesions (cervicitis, CIN1, CIN2, CIN3, and SCC) and *PAX1*^*m*^, Z*NF582*^*m*^, and *PAX1*^*m*^/*ZNF582*^*m*^ were positively correlated (Cochran–Armitage trend test, all *P* < 0.001; Table 1). The positivity rates of *PAX1*^*m*^ and *ZNF582*^*m*^ in all patients were 23.7% and 28.3%, respectively, and their positivity rates in cervical cancer were 100%. The positivity rates for P*AX1*^*m*^/*ZNF582*^*m*^ in cervicitis, CIN1, CIN2, CIN3, and SCC were 17.5%, 18.9%, 35.2%, 65.2%, and 100.0%, respectively.

**Table 1:** Relationship among cervical lesions and *PAX1m*, *ZNF582m*, p16, and Ki67 expression

Biomarker	level	Overall *n* = 371	cervicitis *n* = 103	CIN1 *n* = 95	CIN2 *n* = 71	CIN3 *n* = 89	SCC *n* = 13	*P*_for trend_
p16 (%)	0	112 (30.2)	90 (87.4)	22 (23.2)	0 (0.0)	0 (0.0)	0 (0.0)	< 0.001^&^
	1	62 (16.7)	11 (10.7)	44 (46.3)	6 (8.5)	1 (1.1)	0 (0.0)	
	2	60 (16.2)	1 (1.0)	22 (23.2)	26 (36.6)	11 (12.4)	0 (0.0)	
	3	137 (36.9)	1 (1.0)	7 (7.4)	39 (54.9)	77 (86.5)	13 (100.0)	
Ki67 (%)	0	102 (27.5)	85 (82.5)	16 (16.8)	1 (1.4)	0 (0.0)	0 (0.0)	< 0.001^&^
	1	82 (22.1)	12 (11.7)	45 (47.4)	19 (26.8)	6 (6.7)	0 (0.0)	
	2	105 (28.3)	5 (4.9)	32 (33.7)	40 (56.3)	28 (31.5)	0 (0.0)	
	3	82 (22.1)	1 (1.0)	2 (2.1)	11 (15.5)	55 (61.8)	13 (100.0)	
ΔCp_*PAX1*_ (median [IQR])		18.5 [12.0, 21.2]	19.8 [13.6, 21.8]	19.8 [14.0, 22.1]	18.5 [12.2, 20.9]	13.7 [11.0, 19.8]	8.3 [7.4, 10.1]	< 0.001^#^
*PAX1*^*m*^ (%)	0	283 (76.3)	95 (92.2)	84 (88.4)	56 (78.9)	48 (53.9)	0 (0.0)	< 0.001^$^
	1	88 (23.7)	8 (7.8)	11 (11.6)	15 (21.1)	41 (46.1)	13 (100.0)	
ΔCp_*ZNF582*_ (median [IQR])		13.1 [10.3, 17.6]	13.4 [12.1, 18.5]	13.8 [12.1, 18.2]	13.3 [10.5, 17.8]	9.7 [7.3, 13.6]	2.8 [2.0, 3.2]	< 0.001^#^
*ZNF582*^*m*^ (%)	0	266 (71.7)	92 (89.3)	85 (89.5)	52 (73.2)	37 (41.6)	0 (0.0)	< 0.001^$^
	1	105 (28.3)	11 (10.7)	10 (10.5)	19 (26.8)	52 (58.4)	13 (100.0)	
*PAX1*^*m*^/*ZNF582*^*m*^ (%)	0	239 (64.4)	85 (82.5)	77 (81.1)	46 (64.8)	31 (34.8)	0 (0.0)	< 0.001^$^
	1	132 (35.6)	18 (17.5)	18 (18.9)	25 (35.2)	58 (65.2)	13 (100.0)	
IS (%)	0	85 (22.9)	77 (74.8)	8 (8.4)	0 (0.0)	0 (0.0)	0 (0.0)	< 0.001^&^
	1	30 (8.1)	16 (15.5)	13 (13.7)	1 (1.4)	0 (0.0)	0 (0.0)	
	2	41 (11.1)	6 (5.8)	31 (32.6)	3 (4.2)	1 (1.1)	0 (0.0)	
	3	39 (10.5)	3 (2.9)	26 (27.4)	9 (12.7)	1 (1.1)	0 (0.0)	
	4	43 (11.6)	1 (1.0)	14 (14.7)	22 (31.0)	6 (6.7)	0 (0.0)	
	5	68 (18.3)	0 (0.0)	3 (3.2)	31 (43.7)	34 (38.2)	0 (0.0)	
	6	65 (17.5)	0 (0.0)	0 (0.0)	5 (7.0)	47 (52.8)	13 (100.0)	
IS group (%)	0 ∼ 2	156 (42.0)	99 (96.1)	52 (54.7)	4 (5.6)	1 (1.1)	0 (0.0)	< 0.001^&^
	3 ∼ 4	82 (22.1)	4 (3.9)	40 (42.1)	31 (43.7)	7 (7.9)	0 (0.0)	
	5 ∼ 6	133 (35.9)	0 (0.0)	3 (3.2)	36 (50.7)	81 (91.0)	13 (100.0)	

^&^, Multinomial Cochran-Armitage trend test

^#^, Cuzick trend test

^$^, Cochran-Armitage trend test

### Relationship among p16/Ki67 immunostaining scores, cervical lesions, and ***PAX1/ZNF582*** methylation

**Fig. 2:**
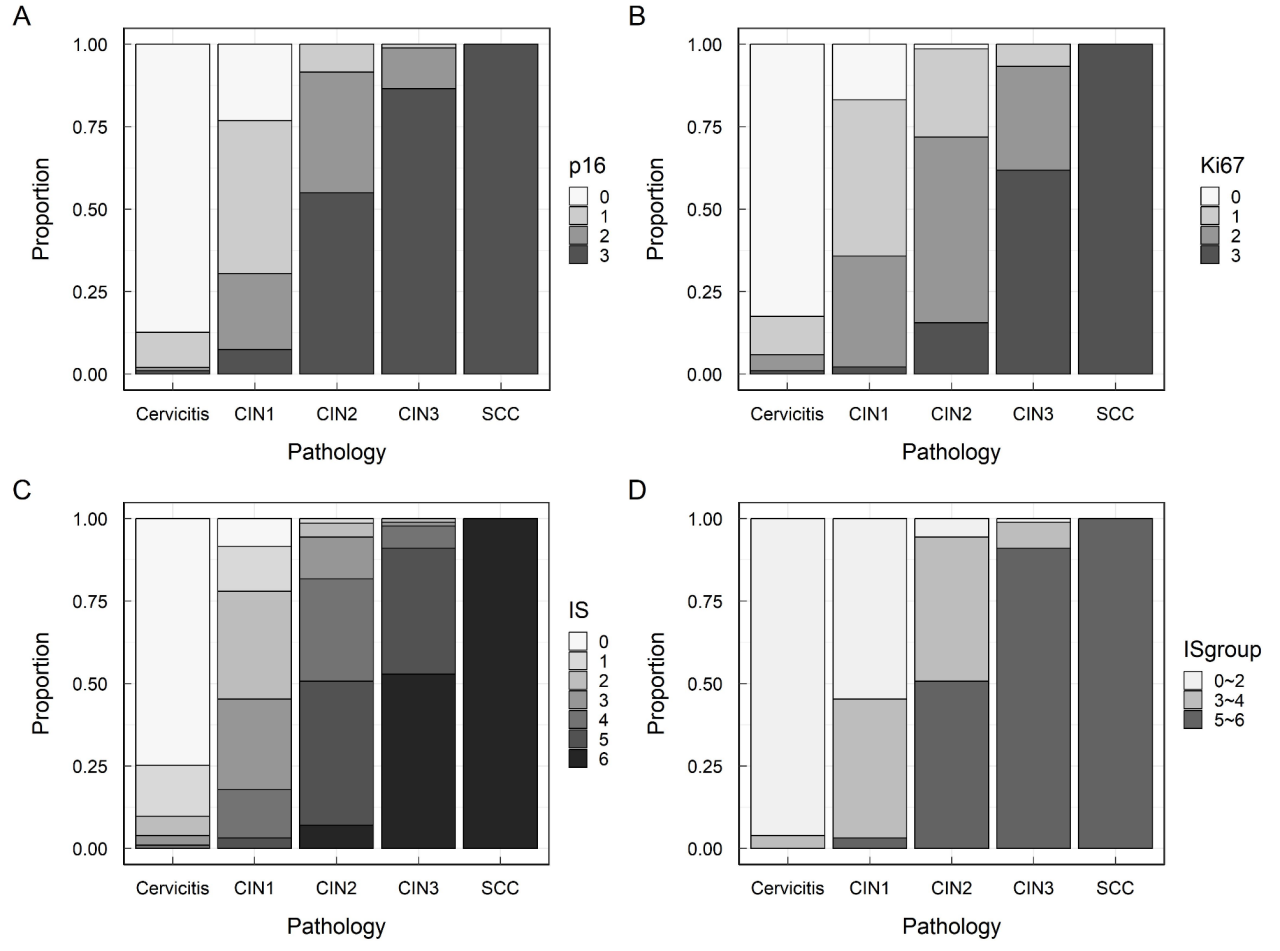
Stack plot of p16/Ki67 immune scores in cervical lesions. A, p16. B, Ki67. C, IS. D, IS group.

The severity of cervical lesions (cervicitis, CIN1, CIN2, CIN3, and SCC) exhibited a clear linear correlation with the immunostaining scores of p16 and Ki67 and the IS (multinomial Cochran–Armitage trend test, all *P* < 0.001; Table 1). The immunostaining scores for p16 and Ki67 were 3, and the IS was 6 in all cervical SCC cases (Fig. 2). In cervicitis, the IS was < 5 in all cases. The ΔCp of *PAX1*^*m*^ and *ZNF582*^*m*^ was statistically significantly different between cases with an IS of 3–4 and 5–6, respectively (all *P* < 0.0001, Fig. 1C and D). The positive rates of *PAX1*^*m*^, *ZNF582*^*m*^, and *PAX1*^*m*^/*ZNF582*^*m*^ increased with an increase in p16, Ki67, and IS (Cochran–Armitage trend test, all *P* < 0.001). The positivity rates of *PAX1*^*m*^/*ZNF582*^*m*^ in cases with p16 and Ki67 immunostaining scores of 3 were 59.9% and 72.0%, respectively. The positivity rate for *PAX1*^*m*^/*ZNF582*^*m*^ was 78.5% in patients with an IS of 6. The positivity rates of *PAX1*^*m*^/*ZNF582*^*m*^ in patients with an IS of 0–2, 3–4, and 5–6 were 17.3%, 26.8%, and 62.4, respectively (Table 2).

**Table 2:** Relationship between p16/Ki67 immunostaining scores and *PAX1*/*ZNF582* methylation

PAX1^m^/ZNF582^m^	Immunostaining score							*P*_for trend_
**p16**	**0**	**1**	**2**	**3**	-	-	-	< 0.001
*PAX1*^*m*^*/ZNF582*^*m*^ (-) (%)	93 (83.0)	48 (77.4)	43 (71.7)	55 (40.1)	-	-	-	
*PAX1*^*m*^*/ZNF582*^*m*^ (+) (%)	19 (17.0)	14 (22.6)	17 (28.3)	82 (59.9)	-	-	-	
Ki67	**0**	**1**	**2**	**3**	-	-	-	< 0.001
*PAX1*^*m*^*/ZNF582*^*m*^ (-) (%)	84 (82.4)	64 (78.0)	68 (64.8)	23 (28.0)	-	-	-	
*PAX1*^*m*^*/ZNF582*^*m*^ (+) (%)	18 (17.6)	18 (22.0)	37 (35.2)	59 (72.0)	-	-	-	
IS	**0**	**1**	**2**	**3**	**4**	**5**	**6**	< 0.001
*PAX1*^*m*^*/ZNF582*^*m*^ (-) (%)	69 (81.2)	26 (86.7)	34 (82.9)	30 (76.9)	30 (69.8)	36 (52.9)	14 (21.5)	
*PAX1*^*m*^*/ZNF582*^*m*^ (+) (%)	16 (18.8)	4 (13.3)	7 (17.1)	9 (23.1)	13 (30.2)	32 (47.1)	51 (78.5)	
IS group	**0 **∼** 2**	**3 **∼** 4**	**5 **∼** 6**	-	-	-	-	< 0.001
*PAX1*^*m*^*/ZNF582*^*m*^ (-) (%)	129 (82.7)	60 (73.2)	50 (37.6)	-	-	-	-	
*PAX1*^*m*^*/ZNF582*^*m*^ (+) (%)	27 (17.3)	22 (26.8)	83 (62.4)	-	-	-	-	

*P*_*for trend*_, Cochran-Armitage trend test

### Subsets of ***PAX1***^***m***^***/ZNF582***^***m***^ and IS in different cervical lesions

Heterogeneity was detected in *PAX1*^*m*^/*ZNF582*^*m*^ and IS among different cervical lesion clusters. Cervical lesions (cervicitis, CIN1, CIN2, CIN3, and SCC) were stratified according to IS and *PAX1*^*m*^/*ZNF582*^*m*^ status (Table 3). There were 36 cases of CIN2 with an IS of 5–6, of which 14 were *PAX1*^*m*^/*ZNF582*^*m*^-positive and 22 were *PAX1*^*m*/^*ZNF582*^*m*^-negative. There were 81 cases of CIN3 with an IS of 5–6, among which 56 were *PAX1*^*m*^/*ZNF582*^*m*^-positive and 25 were *PAX1*^*m*^/*ZNF582*^*m*^-negative. An additional 52 cases of CIN1 had an IS of 0–2, of which 9 were *PAX1*^*m*^/*ZNF582*^*m*^-positive and 43 were *PAX1*^*m*^/*ZNF582*^*m*^-negative. Supplementary Fig. 2 shows the distribution ratios of IS and *PAX1*^*m*^/*ZNF582*^*m*^ in different cervical lesions.

**Table 3:** *PAX1*/*ZNF582* methylation and p16/Ki67 immune score heterogeneity in cervical lesions

Immunoscore group		0 ∼ 2 (*n* = 156)		3 ∼ 4 (*n* = 82)		5 ∼ 6 (*n* = 133)	
*PAX1*^*m*^*/ZNF582*^*m*^ status		*PAX1*^*m*^*/ ZNF582*^*m*^ (-) (*n* = 129)	*PAX1*^*m*^*/ ZNF582*^*m*^ (+) (*n* = 27)	*PAX1*^*m*^*/ ZNF582*^*m*^ (-) (*n* = 60)	*PAX1*^*m*^*/ ZNF582*^*m*^ (+) (*n* = 22)	*PAX1*^*m*^*/ ZNF582*^*m*^ (-) (*n* = 50)	*PAX1*^*m*^*/ ZNF582*^*m*^ (+) (*n* = 83)
Normal (*n* = 103)	n (%)	82 (63.6)	17(63.0)	3 (5.0)	1 (4.5)	0 (0.0)	0 (0.0)
CIN1 (*n* = 95)	n (%)	43 (33.3)	9 (33.3)	31 (51.7)	9 (40.9)	3 (6.0)	0 (0.0)
CIN2 (*n* = 71)	n (%)	3 (2.3)	1 (3.7)	21 (35.0)	10 (45.5)	22 (44.0)	14 (16.9)
CIN3 (*n* = 89)	n (%)	1 (0.8)	0 (0.0)	5 (8.3)	2 (9.1)	25 (50.0)	56 (67.5)
SCC (*n* = 13)	n (%)	0 (0.0)	0 (0.0)	0 (0.0)	0 (0.0)	0 (0.0)	13 (15.7)

## Discussion

In this study, we evaluated the relationship between cervical lesions with various histopathological characteristics and p16/ki67 immunohistochemical staining and *PAX1*/*ZNF582* methylation in HR-HPV-positive women. The abnormal expression of P16 and Ki-67—two commonly used cyclin markers—is associated with CIN and cervical cancer [25]. In this study, we evaluated the relationship between cervical lesions with various histopathological characteristics and p16/ki67 immunohistochemical staining and *PAX1*/*ZNF582* methylation in high-risk HPV-positive women. P16 and Ki67 are two commonly used cyclin markers and their abnormal expression is associated with CIN and cervical cancer [26–30]. Overall, p16 and Ki67 single and double (IS) molecular immunohistochemical staining scores increased with increasing cervical lesion severity. However, the p16 and Ki67 immune scores were 0 and 1 for CIN2 and CIN3, respectively. DNA methylation is an important biomarker that reflects molecular events in the pathophysiology of HPV infection to distinguish cervical lesions that may be at high risk of progression from those that are potentially degenerative [8, 17]. Indeed, many studies have confirmed the clinical performance of DNA methylation for triaging HR-HPV-positive women [23, 31–36]. *PAX1* and *ZNF582* are the most important candidate genes. *PAX1* suppresses the malignant phenotype under oncogenic stress, such as persistent infection by HR-HPV virus. *PAX1* activated a group of phosphatases, including DUSP1, 5, and 6, and inhibited EGF/MAPK signaling to suppress cancer development [37]. Many methylated differential genes were identified in cervical cancer and normal cervix using MeDIP-on-chip technology. One of them, ZNF582, located at chromosome 19q13.43, encodes the zinc finger protein 582, which contains one KRAB-A-B domain and nine zinc-finger motifs that repress transcription of the gene. May be involved in a variety of biological processes related to DNA damage response, proliferation, cell cycle control and tumor transformation. Methylation specific PCR (MSP) methods confirmed that ZNF582 is frequently methylated in CIN3 and more severe lesions [38]. In this study, we found that the ΔCp values of *PAX1* and *ZNF582* methylation decreased with increasing cervical lesion severity (Fig. 1A and B). Furthermore, *PAX1*^*m*^ and *ZNF582*^*m*^ were observed in all cervical cancer cases, whereas approximately 10% were positive for *PAX1*^*m*^ and *ZNF582*^*m*^ in pathological cervicitis (Table 1).

Our study found that the positivity rate for *PAX1*^*m*^/*ZNF582*^*m*^ increased with increasing p16 and Ki67 immunohistochemical scores and IS (Table 2). A similar relationship between p16/Ki67 immunohistochemical staining and *FAM19A4/miR124-2* methylation has been reported in CIN2/3 [19]. High-risk HPV infects the cervical cells of the host and achieves viral multiplication, resulting in CIN (pCIN), which is easily cleared by the immune system. However, HPV DNA integration into host cell DNA is called transforming CIN (tCIN); abnormal expression of E6 and E7 viral proto-oncogenes leads to high-grade lesions and cancer [8, 14]. Additionally, p16/Ki67 co-expression reflects cell dysfunction and is a marker of CIN transformation [8, 17]. Under the action of E6/E7 oncoproteins, the key tumor suppressor proteins p53 and pRb are degraded by proteasomes and p53, as well as the Rb-related transcription factors Sp1 and E2F, bind *DNMT1* promoters to induce its overexpression, and numerous tumor suppressor genes are methylated [39].

Two hypotheses have been proposed regarding the mechanism of cervical precancerous lesion formation. The “progressive progression” model centers on the gradual sequential progression from normal tissue to CIN1, CIN2, and CIN3 [40]. Meanwhile, the “molecular switch” model suggests that DNA methylation serves as a “molecular switch,” allowing CIN3 to evolve directly from HPV-infected normal epithelial cells without progressing through CIN1 and CIN2 [6, 7, 41]. In the current study, nine CIN1 cases had immune scores of 3–4 and were *PAX1*^*m*^/*ZNF582*^*m*^-positive, while three cases had immune scores of 5–6. The presence of CIN1 molecular heterogeneity in these 12 cases suggests the need for closer follow-up every 6 months to avoid short-term progression to CIN3 + and underdiagnosis.

In previous studies, methylation at the CpG sites of *PAX1* progressed to CIN2 + with an HR of 12.62 (95%CI = 1.35, 117.88) over 5 years [18]. Additionally, negative methylation of *FAM19A4*/*miR124-2* is associated with clinical regression of CIN2/3 (odds ratio: 2.72, 95% CI = 1.41–5.26) [19]. Meanwhile, in the current study, among the CIN2/3 cases, four had an IS of 0–2 and were *PAX1*^*m*^/*ZNF582*^*m*^*-*negative, corresponding to a low-grade risk. Thus, for these patients, treatment should be postponed to protect the fertility of women of childbearing age; however, they should be followed up every 12 months. As for the 26 cases with an IS of 3–4 who were *PAX1*^*m*^/*ZNF582*^*m*^-negative, follow-up every 6 months was recommended.

The relationship among the risk of CIN1 progression, CIN2/3 lesion regression, and biomarker lineage expression requires further verification in prospective studies. Accurate prognostic biomarkers can identify women at high risk for CIN, which could improve cervical cancer screening programs. This allows for more precise interventions for lesions with real progression potential, reduces repeat testing, and eliminates the need for treatment in patients with subsided CIN.

The primary strength of this study was the inclusion of different grades of cervical lesions, i.e., cervicitis, CIN1, CIN2, CIN3, and cervical SCC, enabling a comprehensive analysis of the relationship among *PAX1*/*ZNF582* methylation, p16/Ki67 immunohistochemical scores, and cervical lesions. One limitation of the study was the use of paraffin specimens; the processes of DNA extraction and deparaffinization tend to lead to DNA fragmentation, which may affect DNA methylation results. The paraffin specimens were divided into two groups: one for p16/Ki67 immune score analysis and the other for the DNA methylation analysis. Intra-sample differences may have led to a bias in the results. Another limitation is the small sample size and lack of long-term follow-up to assess the risk of progression in low-grade lesions that were *PAX1*^*m*^*/ZNF582*^*m*^- and p16/Ki67-positive and the likelihood of regression in high-grade lesions that were both *PAX1*^*m*^/*ZNF582*^*m*^- and p16/Ki67-negative. However, in this study, immunohistochemistry was performed by a senior technician. The histopathology results were interpreted by two chief physicians, and in the event of inconsistencies, a third pathologist interpreted the results to ensure the accuracy of the results. Overall, we found heterogeneous molecular marker expression in different CIN lesions, providing a new direction for subsequent research on preventing cervical cancer and preventing the overtreatment of cervical lesions.

## Conclusion

A correlation among *PAX1*/*ZNF582* methylation, p16/Ki67 immunohistochemical staining scores, and cervical lesions, with considerable heterogeneity, was observed across CIN cases. This heterogeneity may help clinicians improve personalized management of CIN based on the predicted short-term risk of cancer progression, avoiding missed CIN1 diagnoses and CIN2/3 overtreatment.

## Electronic supplementary material

Below is the link to the electronic supplementary material.

Supplementary Material 1

## Data Availability

The datasets used during the current study are available from the corresponding author on reasonable request.

## Abbreviations

CIN: Cervical intraepithelial neoplasia
HPV: Human papillomavirus
H&E: Hematoxylin/eosin staining
IS: Immune score
*PAX1*^*m*^: *PAX1* methylation
*ZNF582*^*m*^: *ZNF582* methylation
ROC: Receiver operating characteristics curve
