# Supplementary material for: Relationship between p16/ki67 immunoscores and PAX1/ZNF582 methylation status in precancerous and cancerous cervical lesions in high-risk HPV-positive women
Source: BMC Cancer. 2024 Sep 20;24:1171. doi: 10.1186/s12885-024-12920-4 (PMC11414254; doi:10.1186/s12885-024-12920-4)
Supplement: Supplementary file 1 — Supplementary Material 1 [file 12885_2024_12920_MOESM1_ESM.docx]

Supplementary Figure 1.

Supplementary Table 1 Basic patient clinical characteristics

|  | **level** | **n (%)** |
| --- | --- | --- |
| **n** |  | 371 |
| **Age (median [IQR])** | | 42.0 [32.0, 54.0] |
| **Cytology (%)** | NILM | 58 (15.6) |
|  | ASCUS | 70 (18.9) |
|  | LSIL | 32 (8.6) |
|  | ASC-H | 38 (10.2) |
|  | HSIL | 15 (4.0) |
|  | Missing | 158 (42.6) |
| **p16 (%)** | 0 | 112 (30.2) |
|  | 1 | 62 (16.7) |
|  | 2 | 60 (16.2) |
|  | 3 | 137 (36.9) |
| **Ki67 (%)** | 0 | 102 (27.5) |
|  | 1 | 82 (22.1) |
|  | 2 | 105 (28.3) |
|  | 3 | 82 (22.1) |
| **ΔCp *_PAX1_*** (median [IQR]) | | 18.5 [12.0, 21.2] |
| **ΔCp *_ZNF582_*** (median [IQR]) | | 13.1 [10.3, 17.6] |
| **Pathology (%)** | Normal | 103 (27.8) |
|  | CIN1 | 95 (25.6) |
|  | CIN2 | 71 (19.1) |
|  | CIN3 | 89 (24.0) |
|  | SCC | 13 (3.5) |


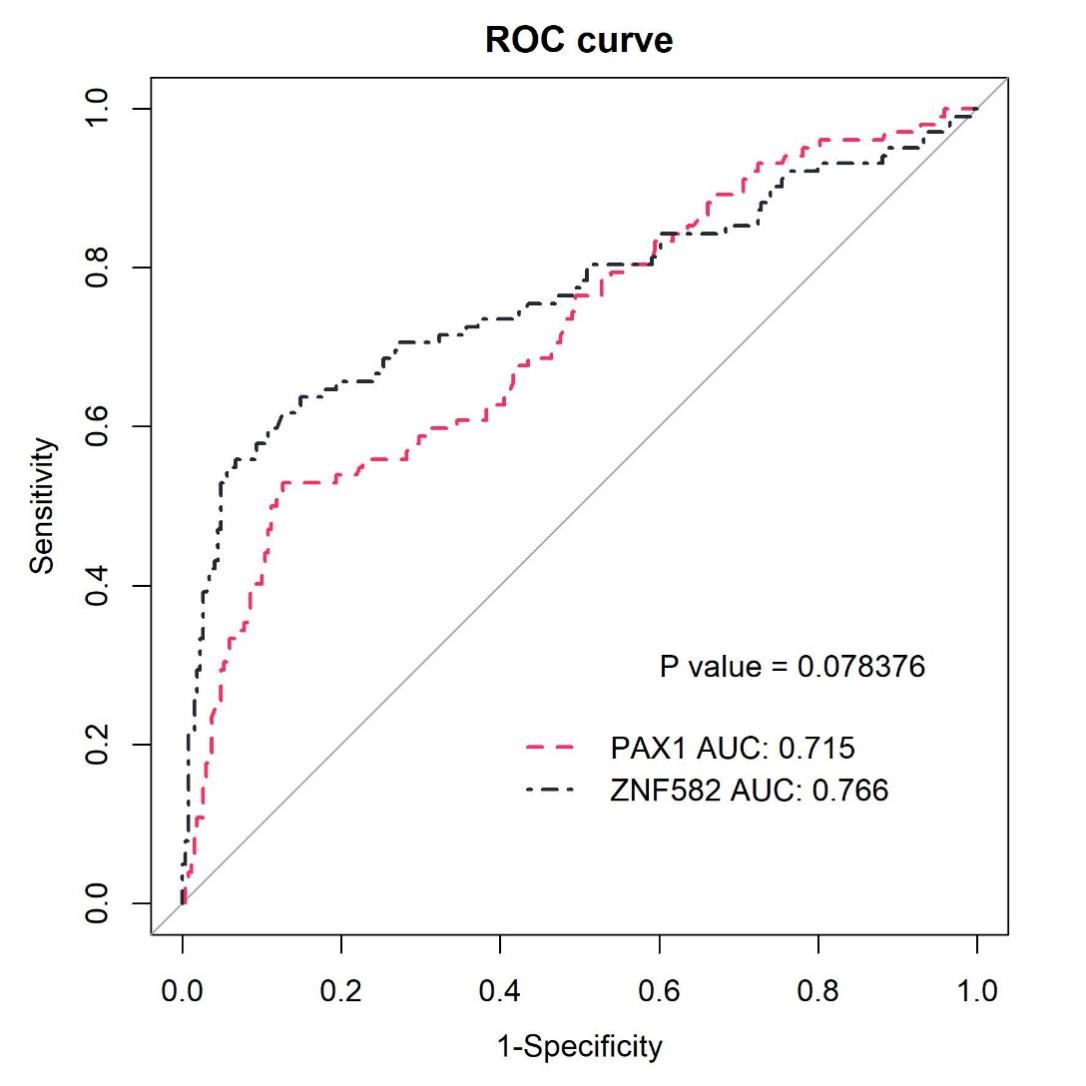


Supplementary Figure 1. ROC curves of *PAX1*/*ZNF582* methylation for CIN3+ detection


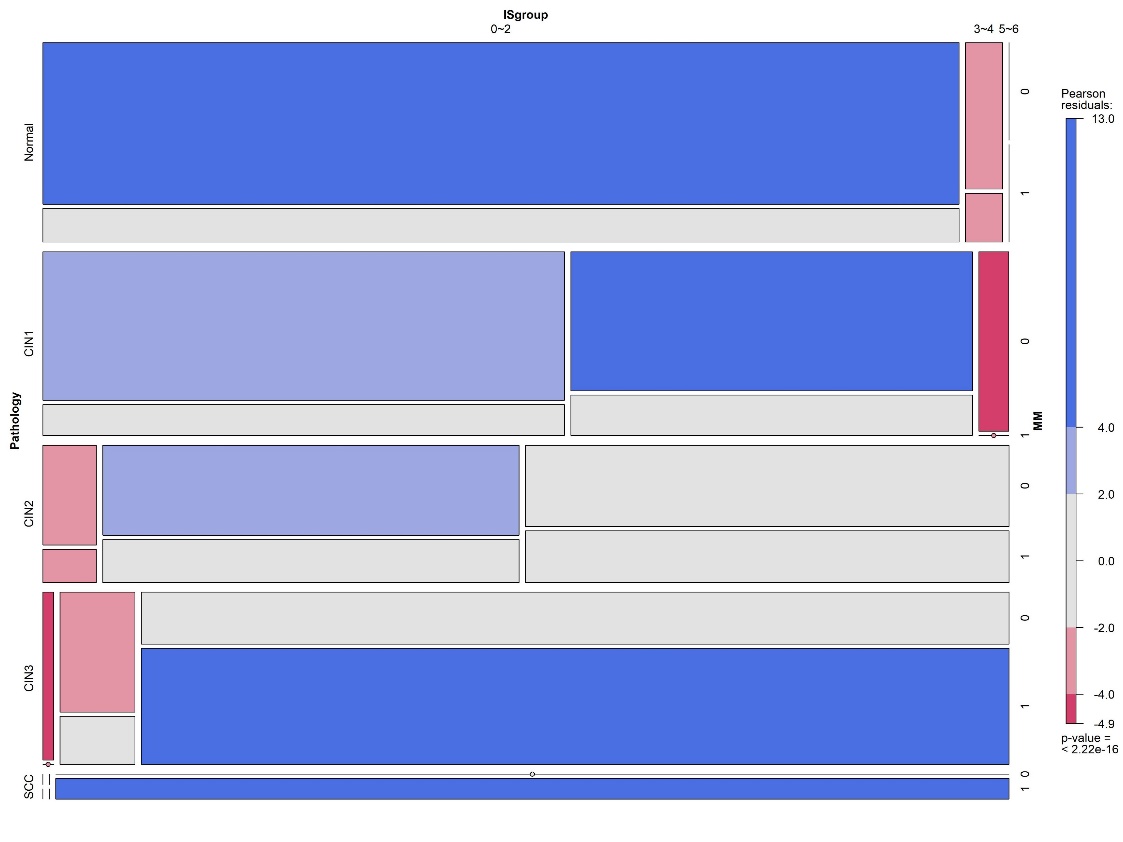


Supplementary Figure 2.
